# Is RAF1 protein from *Synechocystis* sp. PCC 6803 really needed in the cyanobacterial Rubisco assembly process?

**DOI:** 10.1007/s11120-017-0336-4

**Published:** 2017-01-20

**Authors:** Piotr Kolesinski, Malgorzata Rydzy, Andrzej Szczepaniak

**Affiliations:** Laboratory of Biophysics, Faculty of Biotechnology, University of Wroclaw, F. Joliot-Curie 14a, 50-383 Wroclaw, Poland

**Keywords:** RAF1, Rubisco, Assembly chaperones, Carbon fixation, Cyanobacteria

## Abstract

Ribulose-1,5-bisphosphate carboxylase/oxygenase (Rubisco) is responsible for carbon dioxide conversion during photosynthesis and, therefore, is the most important protein in biomass generation. Modifications of this biocatalyst toward improvements in its properties are hindered by the complicated and not yet fully understood assembly process required for the formation of active holoenzymes. An entire set of auxiliary factors, including chaperonin GroEL/GroES and assembly chaperones RbcX or Rubisco accumulation factor 1 (RAF1), is involved in the folding and subsequent assembly of Rubisco subunits. Recently, it has been shown that cyanobacterial RAF1 acts during the formation of the large Rubisco subunit (RbcL) dimer. However, both its physiological function and its necessity in the prokaryotic Rubisco formation process remain elusive. Here, we demonstrate that the *Synechocystis* sp. PCC 6803 strain with *raf1* gene disruption shows the same growth rate as wild-type cells under standard conditions. Moreover, the Rubisco biosynthesis process seems to be unperturbed in mutant cells despite the absence of RbcL-RAF1 complexes. However, in the tested environmental conditions, sulfur starvation triggers the degradation of RbcL and subsequent proteolysis of other polypeptides in wild-type but not Δ*raf1* strains. Pull-down experiments also indicate that, apart from Rubisco, RAF1 co-purifies with phycocyanins. We postulate that RAF1 is not an obligatory factor in cyanobacterial Rubisco assembly, but rather participates in environmentally regulated Rubisco homeostasis.

## Introduction

Ribulose-1,5-bisphosphate carboxylase/oxygenase (Rubisco) catalyzes the first reaction of the Calvin–Benson–Bassham cycle—incorporation of carbon dioxide into a sugar substrate, synthesizing two 3-phosphoglycerate molecules as a product. As this enzyme plays a significant role in carbohydrate and, in a broader range—biomass synthesis, it remains the center of interest in agricultural production and biofuel technology. The catalytic inefficiency of Rubisco, manifested both in the slow rate (3–12 s^−1^; Sage 2002) and possibility of erroneous incorporation of oxygen into RuBP resulting in the formation of toxic phosphoglycolate, makes this enzyme a desirable target for enhancement by directed mutagenesis. However, the establishment of a feasible and easy screening method for promising mutations is still hindered by the complicated and not fully understood Rubisco biosynthesis process, especially for the more complex form I of the enzyme. This form, which is met in eukaryotic photosynthetic organisms as well as in cyanobacteria, is built of eight large (RbcL) and eight small (RbcS) subunits, which have to be properly folded and then assembled in a step-wise manner. An entire set of auxiliary proteins involved in Rubisco biogenesis has been identified and functionally characterized. It is postulated that to achieve a proper tertiary structure RbcL requires the action of chaperonin (chloroplast Cpn60/Cpn20/Cpn10 or prokaryotic GroEL/GroES; Barraclough and Ellis 1980; Goloubinoff et al. 1989). Moreover, before that stage, a specific DnaJ-like factor—Bsd2, found in eukaryotic chloroplasts seems to be crucial during the folding of the N-terminal domain of the large Rubisco subunit (Brutnell et al. 1999; Doron et al. 2014). To form the octameric core of Rubisco, properly folded RbcLs first need to be dimerized, and to achieve this goal the participation of the so-called assembly chaperones is required. These factors enable proper mutual orientation of the hydrophobic surfaces of RbcL monomers which would tend to aggregate without these guiding proteins. Although two such polypeptides have been identified—RbcX and Rubisco accumulation factor 1 (RAF1), their modes of action are slightly different. The central cleft of RbcX recognizes a specific sequence in the C-terminal tail of one RbcL subunit, whereas its apical part provides an additional waypoint for appropriate positioning of the second RbcL subunit (Saschenbrecker et al. 2007; Bracher et al. 2011). RAF1, on the other hand, binds along the RbcL dimer interface, which suggests an alternate way of guiding the arrangement of bilateral large Rubisco subunits (Hauser et al. 2015). Recently, another Rubisco assembly chaperone—RAF2, has been identified. Although its structure has been solved (Wheatley et al. 2014), both its exact physiological function as well as its mode of action remain obscure. However, it may act during the addition of small Rubisco subunits into the octameric core of the enzyme (Feiz et al. 2014).

Although RbcX as well as RAF1-encoding genes are present in both plant and cyanobacterial genomes, the requirement for their participation in Rubisco assembly seems to be kingdom-related. Except for Rubisco from *Synechococcus elongatus* PCC 7942 or its derivatives, where the biosynthesis process seems to be at least partially RbcX-independent (Emlyn-Jones et al. 2006), cyanobacterial holoenzymes rely on this assembly chaperone to reach a properly folded and active state (Onizuka et al. 2004; Li and Tabita 1997). In contrast, there is still no indication of the requirement for RbcX involvement in plant Rubisco assembly, although it may fulfill this function toward cyanobacterial enzymes (Kolesinski et al. 2011). During biosynthesis of plant Rubisco, the assembly machinery instead depends on the RAF1 protein, which is confirmed by the lethality of RAF1-encoding gene disruption in maize (Feiz et al. 2012) as well as the requirement for *Arabidopsis thaliana* RAF1 for proper biogenesis of *Arabidopsis* enzymes in tobacco cells (Whitney et al. 2015). Although the ability of RAF1 to promote prokaryotic RbcL assembly in vitro as well as in *Escherichia coli* is indisputable (Hauser et al. 2015; Kolesinski et al. 2014), the physiological function of this factor in cyanobacterial cells remains unclear.

Here, we report that RAF1 is not required in the biogenesis of cyanobacterial Rubisco from *Synechocystis* sp. PCC 6803. The RAF1 deletion strain of this cyanobacteria shows no significant perturbation in the growth rate or Rubisco amount under standard cultivation conditions. However, under conditions of sulfur depletion, the starvation phenotype appears to be delayed and milder in the Δ*raf1* mutant than in wild-type cells. Also, except for large and small Rubisco subunits, RAF1 co-precipitates with both α and β subunits of phycocyanin during pull-down experiments. We postulate that, in contrast to the plant cell where this factor plays a pivotal role in Rubisco assembly, its cyanobacterial homolog fulfills an auxiliary function participating in environmental condition-dependent regulation of Rubisco homeostasis.

## Materials and methods

### Growth conditions

*Synechocystis* sp. PCC 6803 were cultivated on BG-11 medium (Rippka 1988) at 30 °C under continuous illumination of 50 µE ×  m^−2^ × s^−1^. Environmental stresses were applied to the cultures of OD_730_~1 as described further. For salt stress, BG-11 medium was supplemented with 0.5 M NaCl according to Kanesaki et al. (2002) and cells were grown for an additional 72 h. Oxidative stress was carried out as described by Li et al. (2004)—by the addition of H_2_O_2_ to the medium to a final concentration of 1.5 mM and incubation for a subsequent 30 min. Sulfur depletion was implemented by the replacement of sulfate salts in BG-11 medium by corresponding chlorides of the same concentration. Cells of appropriate OD_730_ were washed and next re-suspended in sulfur-free medium and then cultivated for another 72–144 h. The optical density of all re-suspended cells measured at 730 nm was set to the same value of 0.5. For sulfur repletion, stressed cells after 72, 120, or 144 h were supplemented with MgSO_4_ added to the cultures on BG-11(-S) medium to the final concentration of 75 mg/l, i.e., an equivalent amount of sulfate to that in standard BG-11. After MgSO_4_ supplementation, cultures were carried out for an additional 48 h. To compare growth and pigment absorbance of cells after sulfate repletion, wild-type and mutant cells starved for 144 h were set to the same OD_730_ value of 0.8 and cultivated for another 48 h after addition of MgSO_4_. Before and after sulfate supplementation absorption spectra of wild-type and Δ*raf1* cells were measured.

### Transformation of cyanobacteria

Transformation of *Synechocystis* sp. PCC 6803 cells was performed according to the procedure described by Grigorieva and Shestakov (1992). In short, cells of OD_730_~1 were centrifuged for 10 min. at 3000×*g* at RT and re-suspended in fresh BG-11 medium to OD_730_ of ca. 40. For each 0.5 ml of cell suspension, 50 µg of circular, water-dissolved plasmid was added. The mixture was incubated overnight at RT in darkness with gentle rotation and then propagated on solid BG-11 medium supplemented with 5 mM glucose and 25 µg/ml kanamycin. Colonies appeared after 7 to 10 days. To achieve full segregation of the introduced mutation, cells underwent subsequent passages on BG-11 medium with increasing kanamycin concentrations (from 25 to 50 µg/ml). The genetic homogeneity of the obtained strain was confirmed using PCR with 6803KOFw and 6803KORv primers (sequences are listed in Table 1). Homogenous mutants were further cultivated on BG-11 medium without the addition of either glucose or an antibiotic.

**Table 1:** List of oligonucleotides used in this study

Name	Sequence
6803KOFw	GAAGGCAGGTTACGGTGC
6803KORv	CCTTTAGCTTACTCCAAG
rafSynKOFw	TATGAATTCTGAATAGGAAAAGTTCCAC
rafSynKORv	TATAAGCTTGACGATGGCAAGATGG
pKmFw	AAAGGTTACCGTTAAGGGATTTTGGTCATG
KmRv	AAACCATGGCTTAGAAAAACTCATCGAGC
raf1Fw	AAACATATGACCCATTCCCCTGAATCC
raf1Rv	AAAGAATTCTAATCATCCATTTGCCATGGTTC
raf1NStFw	AAACATATGTGGAGCCACCCGCAGTTCGAAAAAGCGACCCATTCCCCTGAATCC
raf1CStRv	AAAGAATTCCTATTTTTCGAACTGCGGGTGGCTCCATGCATCATCCATTTGCCATG
rbcXFw	AAACATATGTTCATGCAAACTAAGCACATAGC
rbcXRv	AAAGAATTCTTAGGACGGGGGAGAATCGTTG
rbcL6803Fw	CGAATTCGAGCTCGGTACCCATGGTACAAGCCAAAGCAGG
rbcL6803Rv	ATTATTTCTAGAGGATCCCCGGTTTAGAGGGTATCCATGG
rbcL6803*Fw	AAAGAATTCATGGTACAAGCCAAAGCAGG
rbcL6803*Rv	AAACTGCAGTTAGAGGGTATCCATGG
rbcL7942Fw	AAAGGATCCGGGACTGCAGCTTTACAG
rbcL7942Rv	AAATCTAGACCCCAGCGATAGTCAGAGG

### Nucleic acid manipulation

Cyanobacterial genomic DNA was isolated according to the procedure described by Wu et al. (2000). All plasmid vectors were purified using a GeneMATRIX plasmid miniprep DNA purification kit (EURx, Gdansk, Poland).

For construction of *raf1*, deletion plasmid 2357 bp DNA fragment containing the *sll0102* gene with overlaps was amplified using Phusion polymerase (Thermo Fisher Scientific, Waltham, MA, USA), rafSynKOFw and rafSynKORv primers, with *Synechocystis* genomic DNA as a template. Obtained DNA was purified, digested with *Eco*RI and *Hin*dIII nucleases, and cloned into a pTZ57R vector (Thermo Fisher Scientific) digested with the same set of restriction enzymes. The constructed vector was digested with *Bst*EII and *Nco*I enzymes, resulting in the removal of the 657 bp fragment of the RAF1-encoding gene. The deleted fragment was replaced with a kanamycin resistance gene with its own promoter fragment amplified with Phusion polymerase, pKmFw and KmRv primers, and a pET-24b vector (Novagen, Darmstadt, Germany) as a template, which was purified and subsequently digested with *Bst*EII and *Nco*I nucleases. The obtained plasmid pTZ57raf1KO was directly used for *Synechocystis* transformation.

For over-expression of N- and C-terminally Strep-tagged as well as untagged variants of *raf1* gene from *Synechocystis* sp. PCC 6803, DNA fragments were amplified with the use of Phusion polymerase, genomic cyanobacterial DNA, and a suitable set of primers (raf1NStFw and raf1Rv; raf1Fw and raf1CStRv; or raf1Fw and raf1Rv, respectively). The obtained DNA fragments were purified and digested with *Nde*I and *Eco*RI nucleases and cloned into a pET-22b vector (Novagen, Darmstadt, Germany), cut with the same set of enzymes, resulting in plasmids termed pET22raf1NSt, pET22raf1CSt, and pET22raf1. The *rbcX* gene from *Synechocystis* sp. PCC 6803 was amplified as described above with the use of rbcXFw and rbcXRv primers, and cloned into *Nde*I and *Eco*RI sites of a pET-22b vector, resulting in a pET22rbcX plasmid.

For the co-expression experiment, the desired genes were cloned into a pUC18 vector (Thermo Fisher Scientific) or its derivatives. Plasmid pUC18(LR)_6803_ carrying *rbcL* and *raf1* genes from *Synechocystis* sp. PCC 6803 was constructed by subsequent cloning of *raf1* and then *rbcL* genes. The *raf1* gene-containing fragment was digested from pET22raf1 with *Xba*I and *Hin*dIII enzymes and sub-cloned into pUC18 giving a pUC18raf1 plasmid. The *Synechocystis rbcL* gene was amplified as described above with the use of rbcL6803Fw and rbcL6803Rv. After the digestion of the pUC18raf1 plasmid with *Sma*I nuclease, the *rbcL* gene was introduced with a Gibson Assembly Cloning Kit (New England Biolabs, Ipswich, MA, USA). Simultaneously, the *rbcL* gene from *Synechocystis* was amplified with rbcL6803*Fw and rbcL6803*Rv primers and cloned into the *Eco*RI and *Pst*I sites of the pUC18 plasmid resulting in a pUC18rbcL_6803_ vector. To obtain plasmid pUC18L_7942_R_6803_ carrying an *raf1* gene from *Synechocystis* sp. PCC 6803 and an *rbcL* gene from *S. elongatus* PCC 7942, the *rbcL* gene was amplified using rbcL7942Fw and rbcL7942Rv primers and cloned into the *Bam*HI and *Xba*I sites of the pUC18raf1 plasmid. Vector pUC18rbcL_7942_ carrying an *rbcL* gene from *S. elongatus* alone was constructed in a similar manner, but ligation was performed into the pUC18 plasmid instead of pUC18raf1. Vector pUC18L_Te_R_6803_ carrying an *raf1* gene from *Synechocystis* sp. PCC 6803 and an *rbcL* gene from *Thermosynechococcus elongatus* was constructed by sub-cloning an *raf1-*containing fragment of pET22raf1 digested with *Xba*I and *Hin*dIII into the previously described pUC18rbcL plasmid (Tarnawski et al. 2008). pUC18L_Te_X_6803_ carrying an *rbcX* gene from *Synechocystis* sp. PCC 6803 and an *rbcL* gene from *T. elongatus* was constructed by sub-cloning an *rbcX-*containing fragment of pET22rbcX digested with *Xba*I and *Hind*III into pUC18rbcL.

### Protein expression and purification

All proteins were overproduced in *E. coli* Rosetta cells in liquid LB medium supplemented with ampicillin (100 µg/ml) and chloramphenicol (25 µg/ml) at 37 °C. Over-expression of tagged and untagged RAF1 variants was induced by the addition of 0.5 mM isopropyl-β-d -thiogalactopyranoside (IPTG) to the cultures at an attenuance at 600 nm of approximately 0.6, followed by incubation with vigorous shaking for the next 3 h at 37 °C. For induction of RbcL-containing complexes as well as RbcLs alone over-expression, 0.1 mM IPTG was used followed by incubation for the next 5 h. After cultivation, cells were pelleted (2000×*g*) for 10 min at 4 °C, and frozen at −20 °C for further manipulations. All cells were disrupted by sonication in 20 mM Tris pH 8.0, 100 mM NaCl, 1 mM EDTA, 1 mM phenylmethanesulfonyl fluoride (PMSF), and 2 mM β-mercaptoethanol, and the insoluble cell fraction was pelleted by centrifugation at 20000×*g* for 30 min at 4 °C. All RAF1 variants were purified by subsequent ammonium sulfate precipitation, ion exchange chromatography, and size exclusion chromatography. Proteins were desalted with solid ammonium sulfate to 35% (w/v) saturation followed by centrifugation at 20000×*g* for 30 min at 4 °C. The obtained pellets were dissolved in 20 mM Tris pH 8.0, 2 mM β-mercaptoethanol, dialyzed against the same buffer, and then subjected to ion exchange chromatography using a Q-Sepharose column (GE Healthcare, Little Chalfont, UK) pre-equilibrated with 20 mM Tris pH 8.0, 2 mM β-mercaptoethanol. Elution of RAF1 variants was conducted in a 0–500 mM NaCl gradient. Suitable fractions were concentrated with Amicon Ultra Centrifugal Filter Units (Merck Millipore, Billerica, MA, USA) and finally subjected to size exclusion chromatography with a Superdex 75 column (GE Healthcare) pre-equilibrated with 20 mM Tris pH 8.0, 150 mM NaCl, 2 mM β-mercaptoethanol. The RbcL_Te_-RAF1_6803_ complex was purified the same way as RAF1 with the omission of the gel filtration step. The RbcL_Te_-RbcX_6803_ complex and RbcX_6803_ were purified during the same purification procedure. Proteins were precipitated with ammonium sulfate to 30% (w/v) saturation and subjected to purification on a Q-Sepharose column as described for RAF1 proteins. During the elution, the RbcL_Te_-RbcX_6803_ complex-containing fraction is separated from the excess of RbcX protein (unbound RbcX represents ca. 75% of ammonium sulfate precipitated polypeptides). After the purification, all proteins were dialyzed against 10 mM Tris (pH 8.0), 2 mM β-mercaptoethanol. RbcL_8_ from *T. elongatus* was purified as previously described (Kolesinski et al. 2014). Rubisco (RbcL_8_RbcS_8_) from *T. elongatus* was kindly provided by Beata Gubernator (Faculty of Biotechnology, University of Wroclaw, Poland).

### Cyanobacterial extract preparation and RAF1 pull-down

After the cultivation, cyanobacterial cultures were pelleted by centrifugation at 3000×*g* for 10 min at 4 °C. Crude cell extracts were obtained by sonication in 20 mM Tris pH 8.0, 150 mM NaCl, 2 mM PMSF, and 2 mM β-mercaptoethanol followed by centrifugation at 15000×*g* for 15 min at 4 °C. RAF1 pull-downs were performed with the use of protein extracts from wild-type cells. For cell extracts containing 1 mg of proteins, 20 µg of N- or C-terminally Strep-tagged RAF1 was added and pre-incubated for 5 min at RT. After initial pre-incubation, 50 µl of Strep-Tactin Superflow Plus resin (Qiagen, Hilden, Germany) was added and binding was carried out with gentle rotation for 15 min at RT. Resin was washed five times with wash buffer (20 mM Tris pH 8.0, 150 mM NaCl). Bound polypeptides were eluted using wash buffer supplemented with 2.5 mM d-desthiobiotin. PAGE-separated polypeptides were excised from gels, trypsin-digested, and subjected to mass spectroscopy analysis at the Mass Spectroscopy Laboratory of Biophysics and Biochemistry Institute of the Polish Academy of Sciences (Warsaw, Poland).

### Protein electrophoresis and immunoblot analysis

Before electrophoresis, the protein concentration in *E. coli* extracts was measured using Roti-Nanoquant reagent (Carl Roth, Karlsruhe, Germany). For measurement of protein concentration in cyanobacterial extracts, a DC Protein Assay kit (Bio-Rad, Hercules, CA, USA) was employed. Protein separation under denaturing conditions was performed using Tricine-PAGE (Schagger and von Jagow 1987), and for native PAGE a 10% TGX FastCast Acrylamide Kit (Bio-Rad) was employed. Gels were Coomassie-stained except visualization of polypeptides designated for MS analysis, which were silver-stained according to the procedure described by Mortz et al. (2001). For immunodetection, separated proteins were transferred onto a PVDF membrane and incubated with anti-RAF1 (1:2000 dilution for cyanobacterial extracts, 1:20,000 for *E. coli* extracts; raised against purified RAF1 proteins from *Synechocystis* sp. PCC 6803 by BLIRT, Gdansk, Poland), or anti-RbcL antibodies (1:2000 for cyanobacterial extracts, 1:10,000 for *E. coli* extracts; Agrisera, Vännäs, Sweden), and subsequently probed with secondary goat anti-rabbit antibodies conjugated with horseradish peroxidase (Sigma-Aldrich, St. Louis, MO, USA). Chemiluminescence detection was performed with a NOWA Kit (MoBiTec GmbH, Goettingen, Germany) on a ChemiDoc Imaging System (Bio-Rad). Calculation of band density was performed with ImageJ software (Schneider et al. 2012). The significance of results was determined with the Student’s *t* test (*P* = 0.05).

### Rubisco activity assay

A Rubisco carboxylase activity assay was performed as previously described (Gubernator et al. 2008). Equivalents of cyanobacterial cell extracts containing 20 μg of proteins were used for individual measurements.

## Results

**Fig. 1:**
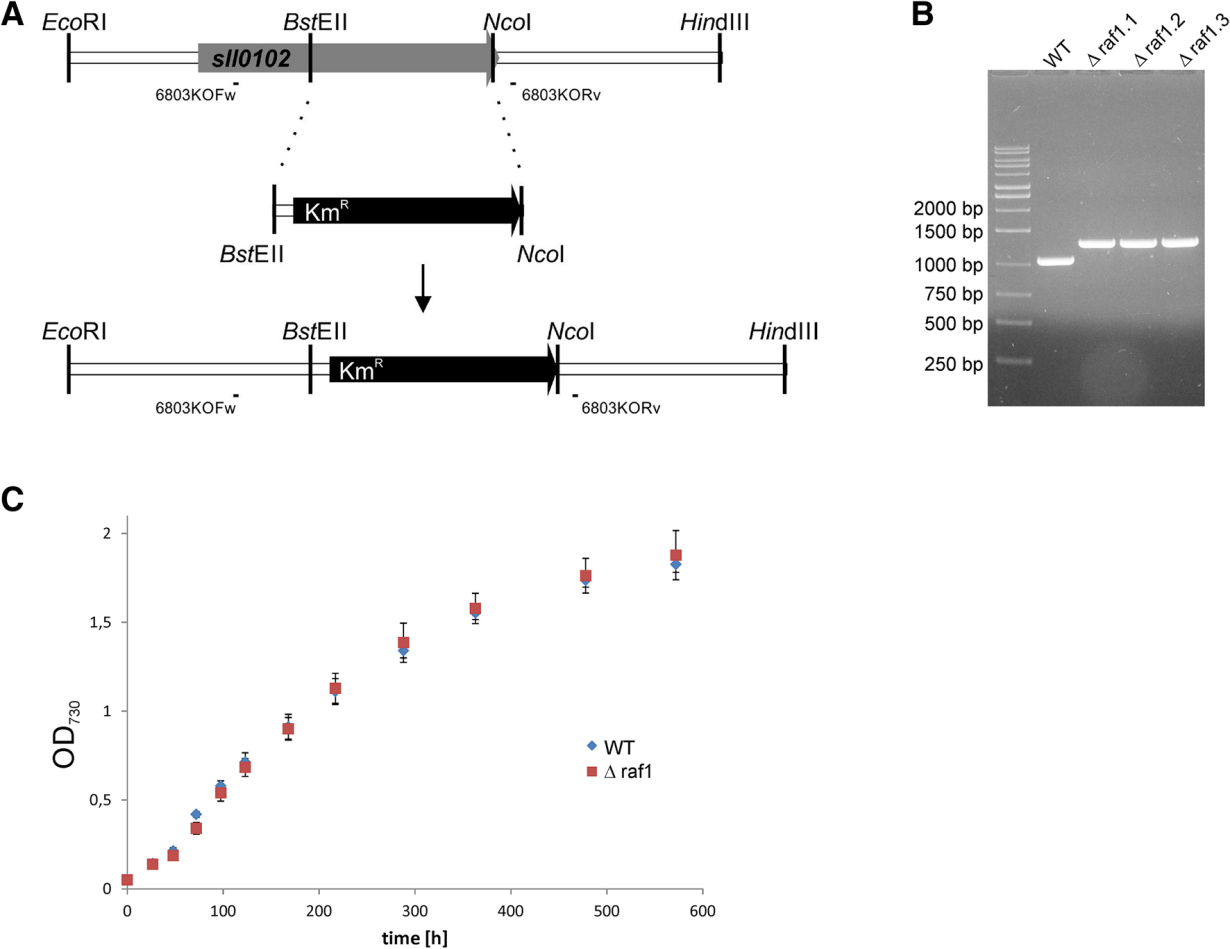
Generation and growth analysis of *Synechocystis* Δ*raf1* mutant. **a** Scheme for insertional inactivation of *sll0102* gene. The 657 bp-long fragment of this gene was replaced with a kanamycin-resistance cassette. A construct was used for transformation of the wild-type cells to generate an *raf1* knock-out mutant. Hybridization positions of primers used for confirmation of the introduced mutation were indicated. **b** Confirmation of mutation introduction (for 3 independent mutant lines) and homogeneity by PCR. **c** Comparison of growth rates of wild-type and Δ*raf1 Synechocystis* cells in standard cultivation conditions monitored by measurement of OD_730_ (mean OD_730_ values for three independent cultures of each strain with standard deviation bars plotted against timescale)

**Fig. 2:**
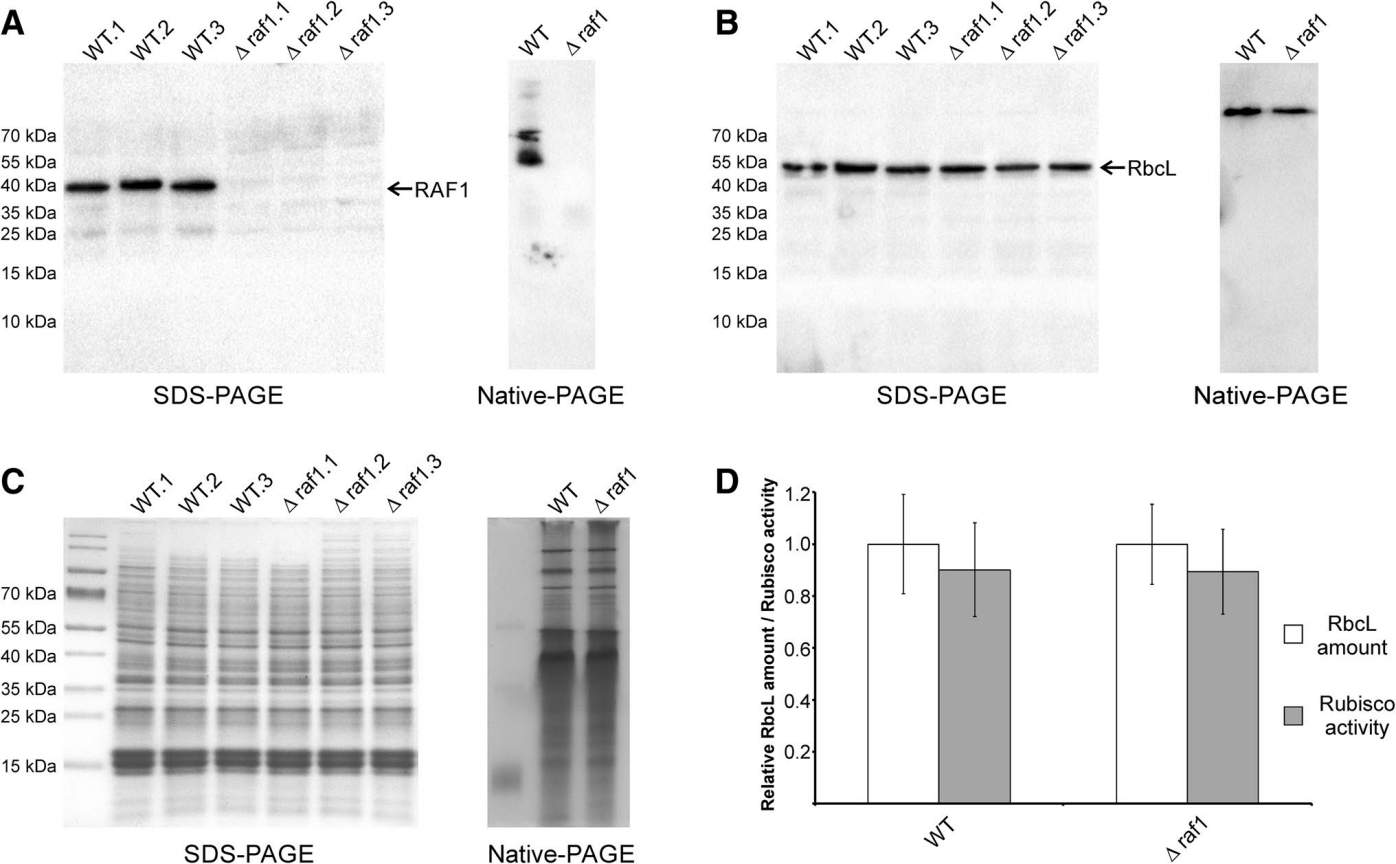
Comparison of RbcL and RAF1 amounts and composition of RbcL- and RAF1-containing complexes in wild-type and Δ*raf1* *Synechocystis* cells cultivated under normal conditions. A total of 20 µg of protein extracts from wild-type and mutant cells were loaded per lane, separated under denaturing (SDS-PAGE; extracts from three independent cultures of wild-type as well as Δ*raf1* strains were used to compare RbcL amounts) or non-denaturing conditions (Native-PAGE), and subjected to immunodetection using anti-RAF (**a**) or anti-RbcL (**b**) antibodies. As a loading control, Coomassie-staining of PAGE-separated extracts was used (**c**). Mean relative RbcL amount calculated by densitometric blot analysis and Rubisco activity in three independent cultures of each strain with standard deviation bars were compared using a bar graph (**d**)

**Fig. 3:**
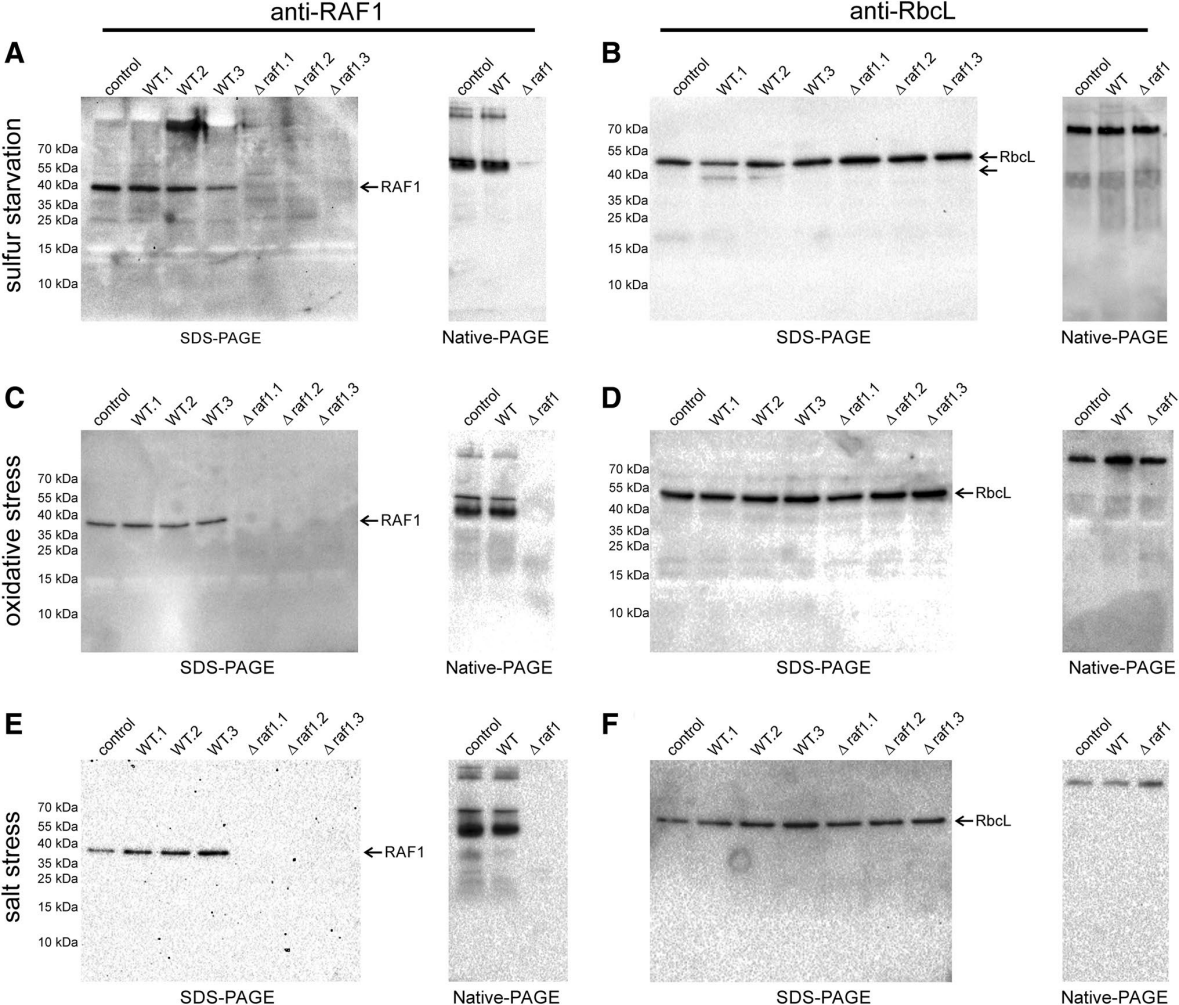
Comparison of RbcL and RAF1 amounts and composition of RbcL- and RAF1-containing complexes in wild-type and Δ*raf1 Synechocystis* cells subjected to sulfur starvation as well as oxidative and salt stress. A total of 20 µg of protein extracts from wild-type and mutant cells were loaded per lane, separated under denaturing (SDS-PAGE; extracts from three independent cultures were used) or non-denaturing conditions (Native-PAGE) and subjected to immunodetection using anti-RAF1 (*left panel* **a** sulfur starvation,** c** oxidative stress,** e** salt stress) or anti-RbcL (*right panel* **b** sulfur starvation,** d** oxidative stress,** f** salt stress) antibodies. As a control, protein extract from untreated wild-type cells was used

To show the physiological function of RAF1 proteins in cyanobacterial cells, we decided to perform *raf1* gene knock-out in a model organism—*Synechocystis* sp. PCC 6803. Disruption of the *sll0102* gene encoding the RAF1 homolog was performed via double crossover with transformation plasmid pTZ57raf1KO, in which ca. 2/3 of the *raf1* sequence was replaced by a kanamycin-resistance cassette (Fig. 1a). The introduced mutation underwent rapid segregation—after only two passages all tested lines showed genomic homogeneity toward *raf1* knock-out, as confirmed by PCR (Fig. 1b). To verify whether RAF1 is a crucial factor in cyanobacterial Rubisco biosynthesis, we compared the growth rates of wild-type and Δ*raf1* cells as well as the amount of RbcL and quality of RbcL-containing complexes in crude extracts from these lines. Disruption of the *raf1* gene did not result in any significant growth perturbations to mutated cyanobacterial lines under standard cultivation conditions (Fig. 1c), although they displayed a slightly increased tendency for adhesion to glass surfaces (i.e., flask bottoms) than wild-type cells (not shown). Immunodetection using anti-RbcL antibodies did not indicate significant differences for RbcL amounts in cell extracts from both wild-type and mutant lines or, in most cases, the presence of modified RbcL-containing complexes (Fig. 2). In addition, the results of Rubisco carboxylase activity measurements remained in agreement with densitometry analyses (Fig. 2d). At the same time, probing with anti-RAF1 antibodies confirmed the existence of at least four different RAF1-containing bands (two low- and two high-molecular weight) in wild-type cells and a lack of these structures in Δ*raf1* mutants. Potential co-localization of RbcL in such high-molecular weight RAF1-containing complexes was barely detectable and only under certain circumstances (see “sulfur starvation” panel in Fig. 3).

**Fig. 4:**
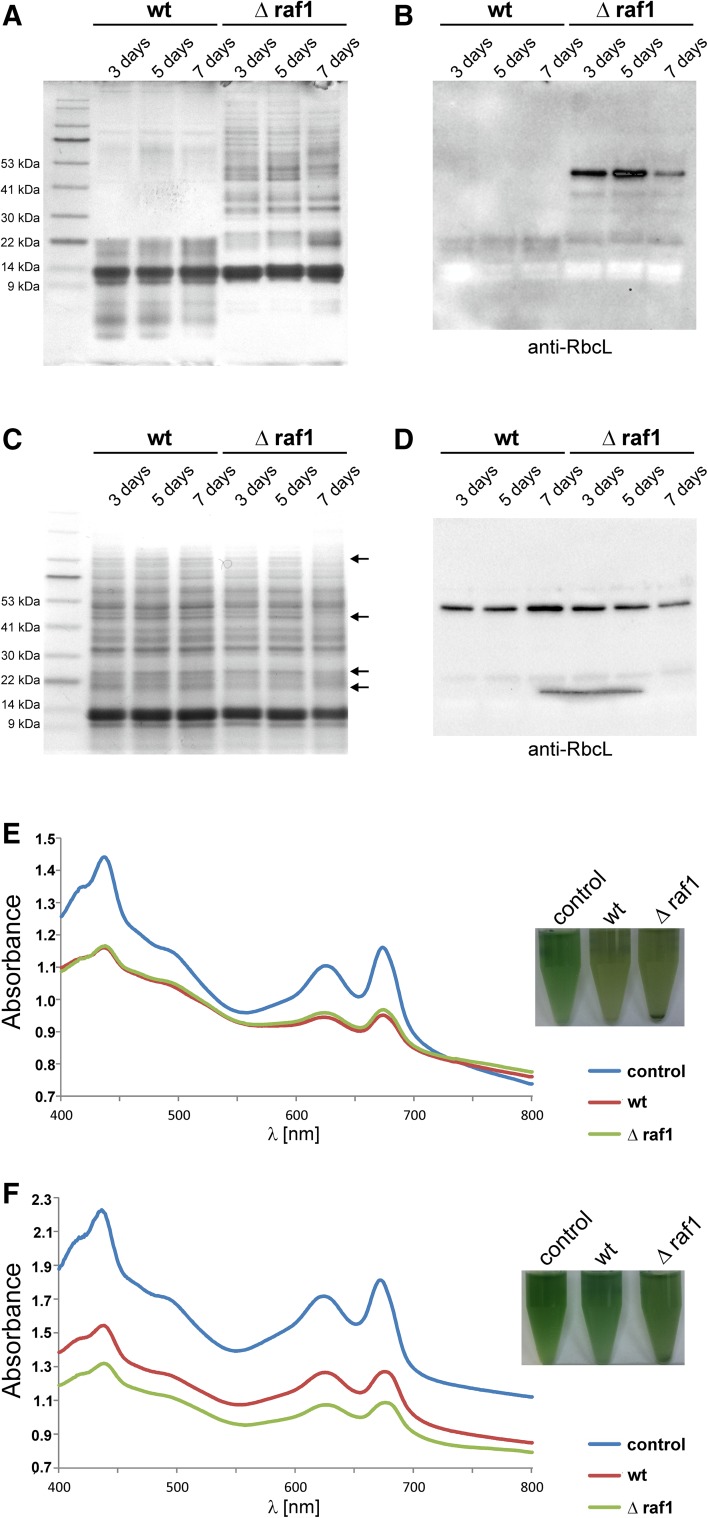
Effect of prolonged exposure to sulfur deprivation and subsequent sulfate repletion for wild-type and Δ*raf1 Synechocystis* cells. Independent cyanobacterial cultures were transferred to sulfur-depleted BG-11 medium and grown subsequently for an additional 3, 5, or 7 days. **a** A total of 20 µg of crude protein extracts from cells collected after treatment were loaded per lane, separated under denaturing conditions, and probed with anti-RbcL antibodies (**b**). The ability of both strains to recover after sulfur depletion was tested by direct supplementation of starved cultures with MgSO_4_ after 3, 5, or 7 days of treatment. **c** A total of 20 µg of crude protein extracts from cells collected after 2-day sulfate re-supplementation were loaded per lane, separated under denaturing conditions, and probed with anti-RbcL antibodies (**d**). Positions of proteins with altered amounts specific for “mild starvation phenotype” of mutant cells are indicated with arrows. **e** Absorbance spectra of wild-type and mutant cells were measured after 7 days of sulfur starvation and following 48 h of sulfate re-supplementation (**f**; control—untreated wild-type cells)

To test the possibility of whether RAF1 is an auxiliary protein required in Rubisco biosynthesis under certain environmental conditions, we decided to grow wild-type and mutated *Synechocystis* cells under conditions causing an increase in *raf1* transcript abundance, as shown by RNA microarray experiments (CyanoEXpress; http://cyanoexpress.sysbiolab.eu/; Hernandez-Prieto and Futschik 2012)—H_2_O_2_-induced oxidative stress and sulfur starvation. In addition, salt stress, which has been reported to negatively affect Rubisco stability (i.e., He et al. 2014), was also implemented. Cells were subjected to abiotic stress, disrupted and their crude extracts PAGE-separated and probed with anti-RbcL and anti-RAF1 antibodies. Among the tested conditions, only sulfur deprivation caused significant differences in the phenotypes of wild-type and Δ*raf1* lines (Fig. 3). While Δ*raf1* mutant seemed to be resistant to sulfur absence in the growth medium, at least on a polypeptide composition level, wild-type cells showed a severe starvation phenotype manifesting in degradation of high-molecular weight proteins, usually after 3 days. At the same time, the amount of RbcL in wild-type cells slightly decreased and its proteolytic products appeared, as judged by immunodetection against SDS-PAGE separated peptides. Observed phenotypes were not strictly repetitive. Alteration of polypeptide composition during starvation was rarely detected for higher density cells (OD_730_ > 1.0); therefore, for further experiments cyanobacterial cultures diluted to OD_730_ of 0.5–0.6 in sulfur-free medium were used. To test the decreased sulfur depletion susceptibility of Δ*raf1* mutants, cultivation on BG-11(-S) medium was prolonged to 7 days. While independent wild-type cultures displayed virtually the same phenotype after 3, 5, and 7 days, a mutant polypeptide composition showed a mild phenotypic effect only after a week (Fig. 4a, b), although RbcL fragmentation was detected earlier. Independent experiments differed in terms of the time of the first observable protein degradation effect—in some cases increased proteolysis was time-shifted to 5 days. However, comparison of wild-type and mutant cells growing simultaneously always resulted in the same pattern—Δ*raf1* never exhibited severe polypeptide fragmentation and its starvation phenotype appearance was always delayed when compared to wild-type culture. To test the survivability of starved cyanobacteria, cells after 3, 5, and 7 days of treatment, respectively, were supplemented with sulfate and left for an additional 2 days of cultivation. A mild starvation phenotype of mutant cells manifested in slightly altered polypeptide composition, and lowered Rubisco amount was still visible after two days of sulfate implementation indicating limited adaptive abilities of *Synechocystis* Δ*raf1* (Fig. 4c, d). Moreover, mutant cells subjected to 7 days of treatment exhibited decreased growth rate in comparison to wild type after sulfate repletion (Fig. 4e, f). In addition, formation of fast-sedimenting cell aggregates was observed for starved Δ*raf1* strain but not for wild type (Fig. 4e).

**Fig. 5:**
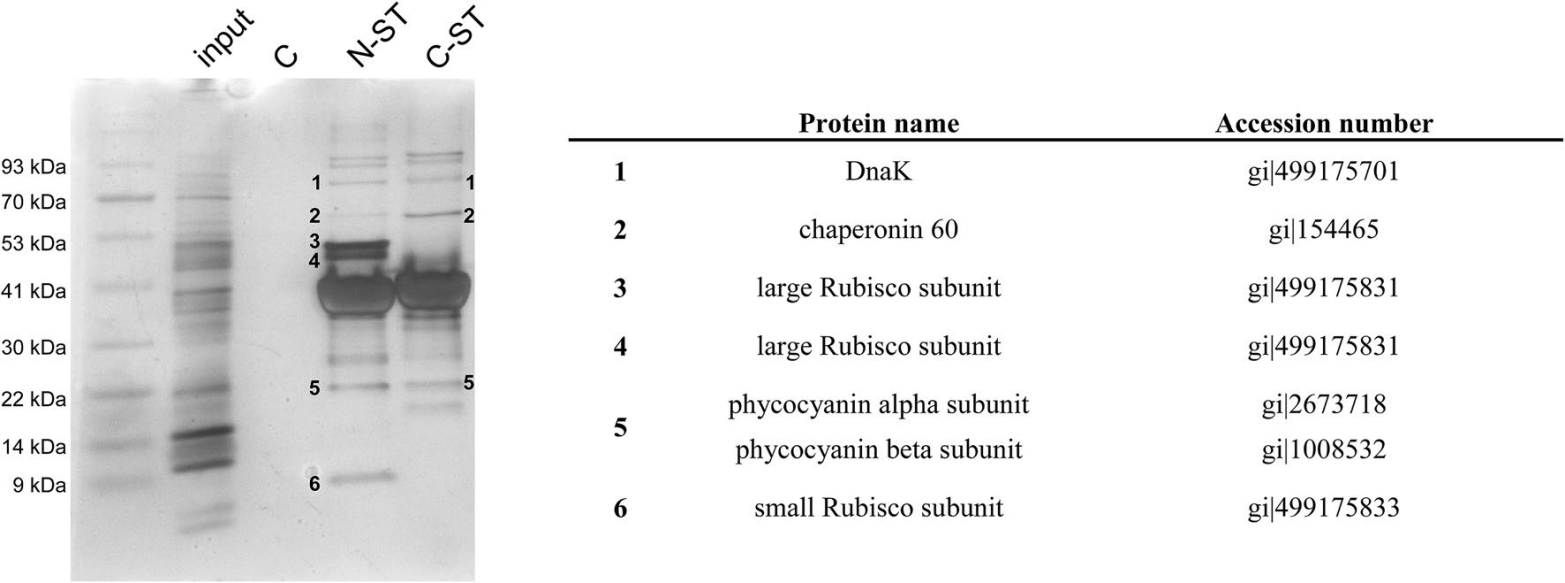
Identification of putative RAF1-interacting proteins in cyanobacterial cells. Crude protein extracts from *Synechocystis* sp. PCC 6803 were incubated with N- (N-ST) or C-terminally (C-ST) labeled RAF1 and subjected to a pull-down experiment using Strep-Tactin Superflow Plus resin. As a control, cell extracts without the addition of labeled RAF1 were used (C). Eluted proteins were separated under denaturing conditions and silver-stained. Separate protein bands were excised from gel and their polypeptide composition was identified by mass spectrometry. Polypeptides which are not RAF1 proteolysis or aggregation products are indicated with subsequent numbers and specified in the table

We decided to verify whether cyanobacterial RAF1 is a part of the Rubisco degradation pathway instead of its assembly. To test this hypothesis, RAF1 pull-down was performed to find RAF1 interactors with indication to chaperones or proteolytic enzymes (e.g., Clp proteases). Since the standard immunoprecipitation approach provided low quality results due to suboptimal yield and high background, N- and C-terminally Strep-tagged RAF1 were added externally to cyanobacterial crude extracts. After elution of tagged RAF1 with potentially bound polypeptides, samples were SDS-PAGE separated and specific protein bands were identified by mass spectroscopy. Apart from Rubisco (both large and small subunits), RAF1 co-purifies with Cpn60, DnaK2, and a relatively high amount of phycocyanin (α and β subunits; Fig. 5). No proteases or unfoldases were found among the identified putative interactors. In addition, C-terminally tagged RAF1 from *Synechocystis* sp. PCC 6803 does not co-purify with Rubisco, suggesting that exposition of its C-terminus may be crucial for interaction with RbcL.

**Fig. 6:**
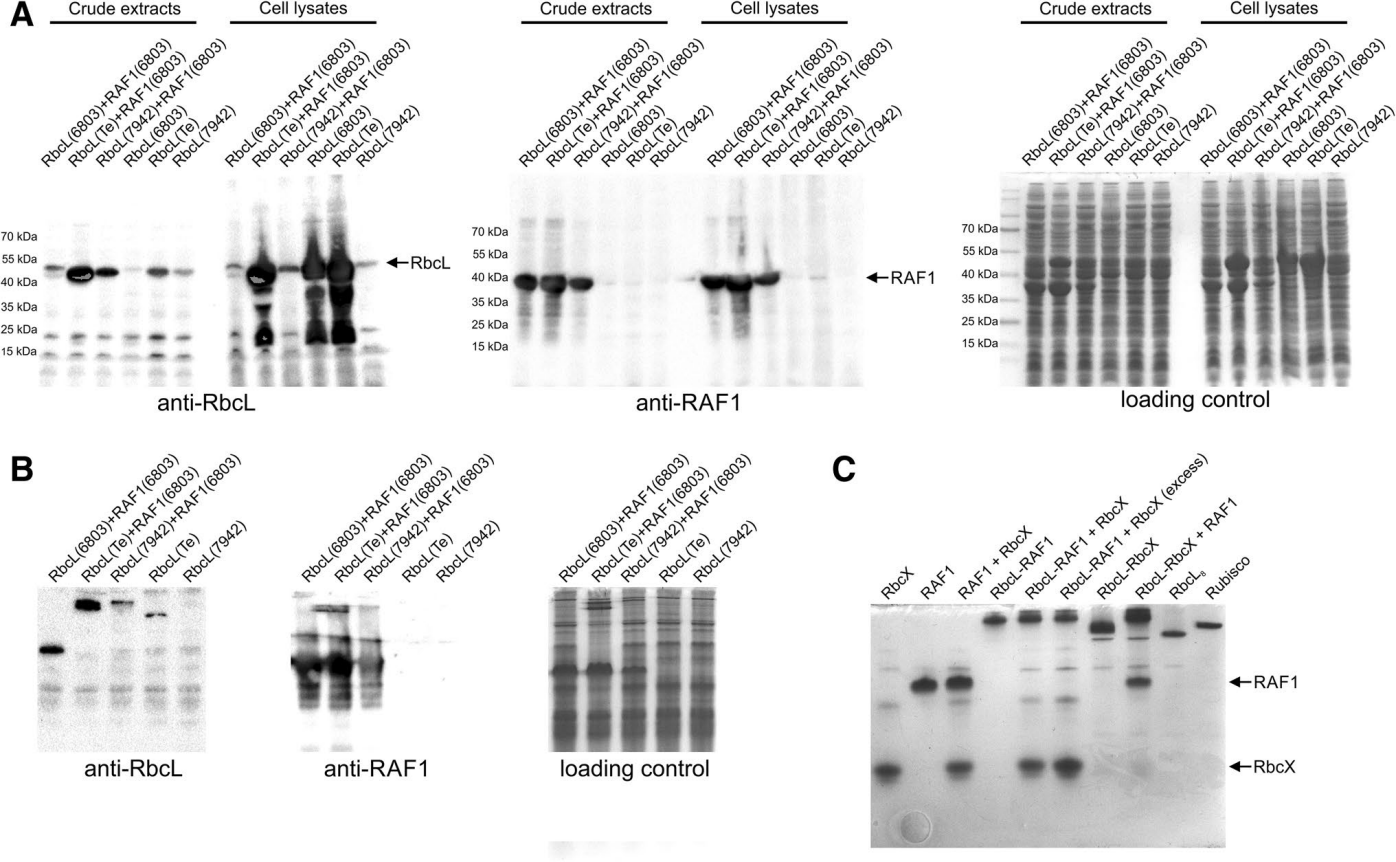
Determination of RAF1 chaperone activity toward cyanobacterial RbcL. **a** Co-expression of *raf1* gene from *Synechocystis* sp. PCC 6803 with *rbcL* from *Synechocystis* sp. PCC 6803 (6803), *Thermosynechococcus elongatus* (Te) or *Synechococcus elongatus* PCC 7942 (7942) in *E. coli* cells. As a control, cells expressing *rbcL* genes alone were used. 20 µg of crude extracts as well as cell lysates from *E. coli* cells were separated under denaturing conditions (SDS-PAGE) and subjected to immunodetection using anti-RbcL or anti-RAF1 antibodies (**a**). Extracts with confirmed soluble RbcL presence were additionally separated under non-denaturing conditions (Native-PAGE) and probed with anti-RbcL or anti-RAF1 antibodies (**b**). **c** Identification of mutual RAF1-RbcL-RbcX interactions. A total of 2.5 µg (or 5 µg in lanes where “excess” is indicated) of RAF1_6803_, RbcX_6803_ proteins as well as RbcL_Te_–RAF1_6803_ or RbcL_Te_–RbcX_6803_ complex were loaded either directly or after pre-incubation with its putative interactor on non-denaturing gel and separated. As a control for complex migration, RbcL_8_ and Rubisco (RbcL_8_RbcS_8_) from *T. elongatus* were used. Positions of unbound RbcX and RAF1 are indicated with arrows. Addition of RAF1 resulted in an upshift of the RbcL_Te_–RbcX_6803_ complex, whereas no substitution of RAF1 in RbcL_Te_–RAF1_6803_ by RbcX or direct interaction between RAF1 and RbcX was observed

To ensure that RAF1 homologs from *Synechocystis* sp. PCC 6803 fulfill the same function during Rubisco assembly *in vitro* and in *E. coli* cells as its counterparts from *T. elongatus* and *S. elongatus* sp. PCC 7942, a Rubisco biosynthesis assay was performed in bacterial cells. An *raf1* gene from *Synechocystis* was co-expressed in *E. coli* with an RbcL-encoding gene from the same organism as well as *rbcL*s from *T. elongatus* and *S. elongatus*. The obtained crude extracts and cell lysates from *E. coli* were separated using SDS-PAGE and subsequently subjected to immunodetection with anti-RbcL and anti-RAF1 antibodies (Fig. 6a). Cell extracts with confirmed soluble RbcL presence were additionally separated under non-denaturing conditions and probed with anti-RbcL or anti-RAF1 antibodies. *Synechocystis* RAF1 promoted folding of its own RbcL resulting in formation of a complex exhibiting a migration pattern similar to those reported for *T. elongatus* (Kolesinski et al. 2014), or *S. elongatus* PCC 7942 (Hauser et al. 2015) RbcL_2_-RAF1_2_ (Fig. 6b). In addition, it effectively showed the same function toward RbcLs from two other cyanobacteria (*T. elongatus* and *S. elongatus* PCC 7942). However, in these cases only larger complexes resembling those identified previously as RbcL_8_–RAF_8_ (Hauser et al. 2015) were observed. Interestingly, spontaneous formation of a presumed octamer was observed for RbcL from *T. elongatus* alone. No such effect was visible for *S. elongatus* RbcL, despite its confirmed foldability. Unfortunately, RbcL_6803_–RAF1_6803_ expression did not scale up to preparative volumes, therefore making it impossible to obtain a purified complex. However, it was possible to over-express in *E. coli* and purify stable complexes containing RbcL from *T. elongatus* with RAF1 or RbcX from *Synechocystis*; therefore, we decided to use this to verify whether there is a probability of the subsequent action of RAF1 and RbcX. The purified RbcL_Te_–RAF1_6803_ complex was mixed with RbcX_6803_ and RbcL_Te_–RbcX_6803_ complex with RAF1_6803_, incubated for 10 min at room temperature and subjected to native-PAGE. RAF1_6803_ and RbcX_6803_ were also mixed in the same manner to verify occurrence of their putative interaction. While RAF1 readily bound to RbcL–RbcX complex, RbcX was not able to replace RAF1 from RbcL_Te_–RAF1_6803_, even in a two-fold mass excess. No direct interaction between RAF1 and RbcX was observed using the described approach (Fig. 6c).

## Discussion

Since plants as well as many cyanobacteria contain both RbcX- and RAF1-encoding genes, it is unclear whether the two proteins participate during the same Rubisco assembly cycle or whether they mainly rely on one of these factors, whereas the function of the second is evolutionary obstructed. Such a hypothesis has mainly been directed toward plant Rubisco chaperones. There is unequivocal evidence for the requirement of RAF1 participation in plant holoenzyme biosynthesis (Feiz et al. 2012; Whitney et al. 2015), which is also supported by the confirmed presence of this polypeptide in the chloroplast proteome (Friso et al. 2010). In contrast, RbcX on a protein level has never been directly identified in plant extracts, although *rbcX* genes are transcribed in eukaryotic cells (Kolesinski et al. 2011; Ramegowda et al. 2014). On the other hand, RbcX was, for a long time, the only known prokaryotic Rubisco assembly chaperone and no performed cyanobacterial holoenzyme assembly assays have ever indicated the need for additional specialized factors (Liu et al. 2010; Mueller-Cajar and Whitney 2008; Larimer and Soper 1993).

Our results suggest that RAF1 from *Synechocystis* sp. PCC 6803 is not required for Rubisco assembly from this organism. Disruption of the *raf1* gene does not seem to affect cell viability, growth, or either the amount or the formation of Rubisco. However, in contrast to plant RbcX, this particular polypeptide is detectable by western blot in cyanobacterial extracts. Moreover, similar to maize cells (Feiz et al. 2012) or in vitro assays (Kolesinski et al. 2014; Hauser et al. 2015), its various oligomeric states have been observed. On the other hand, there are published examples of situations where deletion of particular chaperone did not result in obvious phenotypes in cases of the existence of additional factors with overlapping specificity. For example, disruption of the DnaK- or trigger factor-encoding gene alone in *E. coli* cells does not have any observable effect in terms of cell growth or protein folding (Deuerling et al. 1999). However, combined *ΔdnaK*/Δ*tig* mutation causes cell lethality. To fully understand the function and, if it exists, reciprocal dependence of RbcX and RAF1 in Rubisco biosynthesis, it would be necessary to generate a double *ΔrbcX*/Δ*raf1* mutant of cyanobacterial cells. Since direct *rbcX* insertion, such as that described by Onizuka et al. (2004) for *Synechococcus* sp. PCC 7002, or its replacement by antibiotic resistance cassette may have a downstream effect on *rbcS* expression, it could be necessary to introduce clean and stable *rbcL-rbcS* in place of an existing Rubisco operon.

As RAF1 obviously was unnecessary for cell viability under standard conditions, there is a possibility that its demand could be triggered by certain external stimuli. Among the tested stress conditions, the only differences between wild-type and mutant cells were observed during sulfur starvation, whereas prolonged oxidative stress exposition caused massive protein degradation in both types of cells (results not shown). However, sulfur depletion-induced phenotypes of wild-type and Δ*raf1* cells seem to be peculiar, given the fact that RAF1 was expected to be a Rubisco assembly chaperone and/or fulfill a Rubisco-protective function. On the other hand, cyanobacterial RAF1 as a Rubisco-binding protein could participate in the holoenzyme degradation pathway. Plant Rubisco has been reported to be proteolyzed in the face of demand for amino acids during leaf senescence or nitrogen starvation periods (Feller et al. 2008). Ribulose-1,5-bisphosphate carboxylase/oxygenase degradation has also been observed during sulfur starvation for common duckweed (*Lemna minor*), without causing the death of this organism (Ferreira and Teixeira 1992). Since the cyanobacterial enzyme contributes to only about a few percent of total cellular protein mass due to the existence of a carbon concentrating mechanism (Raven 2013), it is unlikely that it can be used as a primary amino acid source, in contrast to its plant counterpart. However, sulfur requirement- and/or senescence-activated Rubisco proteolysis could be a signal for degradation of high-molecular weight polypeptides. Whether RAF1 actively participates in such a process remains unclear, since pull-down experiments do not identify as RAF1 interactors any expected proteolytic enzymes, such as Clp (Olinares et al. 2011) or HtrA/Deg (Cheregi et al. 2016) proteases, but rather chaperones—DnaK2 or Cpn60. On the other hand, RAF1 seems to interact with both full-length and slightly truncated RbcL forms, as judged by silver staining and mass spectrometry analysis. The latter has previously been reported to be incorporated into the plant holoenzyme during simultaneous action of putative cysteine endopeptidase (Thoenen et al. 2007). In theory, cyanobacterial RAF1 could be involved in a similar process by incorporation of non-functional subunits into the holoenzyme, “labeling” such structures for degradation.

Another explanation for observed sulfur depletion phenotypes may be RAF1 involvement in control machinery related to the Rubisco biosynthesis process. Recently, Klotz et al. (2016) described a highly orchestrated program accompanying long-term nitrogen starvation-induced chlorosis of *Synechocystis* cells and resuscitation from this state caused by nitrogen source addition. In face of nutrient absence, cyanobacteria switch their metabolism from photosynthesis to glycogen storage, which is accompanied by photosynthetic enzyme degradation. Upon nitrate re-supplementation, cells suppress residual photosynthetic activity, and activate glycogen degradation mechanisms to re-switch to photosynthesis and vegetative growth after 48 h. This sophisticated mechanism is employed to face long-term starvation periods as well as a colonization of new environments. In the absence of macro- or micronutrients, cells have to perform some sort of resource allocation, manifesting in degradation of selected polypeptides and/or photosynthetic pigments, to adapt to new conditions or to survive unfavorable periods (Fraser et al. 2013; Kiyota et al. 2014). Therefore, the lack of severe polypeptide fragmentation observed in Δ*raf1* cells could result from dysfunction of mechanisms switching cell metabolism to alternate paths of nutrient acquisition. A similar, although manifesting mainly in perturbed pigment degradation, effect has previously been observed for non-bleaching Δ*nblA* cyanobacterial mutants under nitrogen (Kiyota et al. 2014) or sulfur depletion (Yu et al. 2015). Although the Δ*raf1* strain could maintain its standard proteome for a longer period under sulfur starvation, prolonged exposure to stress stimuli could eventually lead to cell death due to its decreased adaptive abilities as observed for sulfate-repletion experiments.

Co-expression of *Synechocystis raf1* with an *rbcL* gene from the same organism resulted in formation of a complex with a migration pattern resembling that of RbcL_2_–RAF1_2_ in opposition to higher molecular weight mixed complexes (RAF1 from *Synechocystis* with RbcL from either *T. elongatus* or *S. elongatus*). No presence of RbcL in such (RbcL_2_–RAF1_2_) structures was confirmed for cyanobacterial extracts, probably due to their low amount—below the level of detection of anti-RbcL antibodies, although the position of some RAF1-containing complexes during native-PAGE separation might indicate their presence. RbcL_2_–RAF1_2_, if it is not an artifact observable only in vitro and in *E. coli* cells, might be an important control point rather than a Rubisco assembly intermediate. Our previous report indicated that disassembly of an RbcL octamer by RAF1 is preferred over RbcS addition to RbcL_2_–RAF1_2_ (Kolesinski et al. 2014). Therefore, faced with RbcS absence resulting from its proteolytic degradation or translational arrest, RbcL octamers could be dissociated by RAF1. Whether RbcL_2_–RAF1_2_ could actively trigger transcriptional activation of various proteases or subsequent proteolytic events are the result of a cascade initiated by, e.g., a decline of Calvin–Benson–Bassham cycle metabolites remains unknown. The existence of such regulatory mechanisms, even if not involving RAF1, may be indicated by the presence of RbcS- (but not RbcL) specific protease HhoA in *Synechocystis* cells (Tam et al. 2015). To identify additional RAF1-interactors that could be involved in such putative control machinery, it would be necessary to obtain the *Synechocystis* mutant expressing the tagged variant of RAF1. When an exogenous protein is added to the cell extracts, a non-physiological interaction may be observed. In addition, some weak interaction might be overlooked due to the high level of peptides resulting from RAF1 fragmentation or simply because of the absence of some interactors which are only expressed under certain environmental conditions.

Surprisingly, in contrast to our previously reported results (i.e., Kolesinski et al. 2014), we were able to observe spontaneous formation of an *T. elongatus* RbcL octamer in the absence of any assembly chaperones in *E. coli* cells. This particular effect might result from the quality of water used for LB medium preparation (MiliQ vs. previously used distilled water), since when our standard approach was employed, Rubisco from *Synechocystis* sp. PCC 6803 was also not foldable in *E. coli* cells, when expressed with either RbcX- or RAF1-encoding genes. Although RAF1 promotes or significantly improves cyanobacterial RbcL folding, an experimental approach employing easily foldable Rubisco from *T. elongatus* (Kolesinski et al. 2014) or *S. elongatus* PCC 7942 (Hauser et al. 2015) for assembly chaperone analysis may falsify the true physiological function of the latter, despite its feasibility in the study of the mechanisms of action of these proteins. Therefore, RAF1 can act as a Rubisco assembly chaperone in *E. coli* because of its RbcL-stabilizing properties, although this may not necessarily be its proper purpose in cyanobacterial cells. Moreover, its obligatory chaperone function might be acquired during evolution and be revealed only in the plant cell. Various functions fulfilled by protein orthologs from different photosynthetic organisms are not uncommon and their examples have already been described in the literature (Belcher et al. 2015). *Escherichia coli* DnaK/DnaJ or GroEL/GroES systems may additionally mimic specialized cyanobacterial (but not eukaryotic) Rubisco-interacting proteins at pre-assembly Rubisco biogenesis stages. The existence of such particular factors as interacting with RbcL nascent chain Bsd2 (Doron et al. 2014) or specialized Cpn60 subunits required for rice RbcL folding (Kim et al. 2013) has been confirmed for eukaryotic cells.
